# Turning over New Leaves

**Published:** 1890-08-30

**Authors:** 


### Turning Over New Leaves.

### Home Doctoring.*

Dr. Mackenzie having found a girl suffering from typhoid in a remote country district of Scotland, where she could only be seen by a doctor once a week, determined to brave the wrath of the prudes of his profession and publish this book on home medicine. The book is alphabetically arranged, clearly written, and supplied with an excellent index; it will undoubtedly be more or less useful to such of the public as dwell in remote regions where doctors are few and far between. The most original feature of the book is the use of all the quaint unprofessional titles for various diseases, such as Barbadoes Leg, Baker's Itch, and Child-Crowing. Of course, this is most useful for such readers as the book is written for.

### The Deep Sea Fishermen.†

This is a very favourable specimen of the best form of what may be called appeal literature. The author, assured of the great benefit that has been effected in a good cause by energetic and conscientious action, endeavours by means of a simple and yet telling narrative to enlist public sympathy still further with one of the most interesting and deservedly popular of recent philanthropic movements. Since the year 1881, when attention was first directed to the dissolute and neglected condition of the North Sea trawlers, the Mission to Deep Sea Fishermen has developed and prospered beyond the expectations of the most sanguine. Indeed, the success has been so great that we might be disposed to look with some little misgiving to its future, were we not aware of the sagacity and enterprise of the leaders of the movement who, relying on its claims for support, are now extending its benefits beyond the German Ocean, and rendering aid to fishermen in other seas. The contents of this interesting volume fall into three main divisions. Mr. Gordon first describes the smacksman of olden times, his habits, and his peculiar temptations and enemies. He next gives a history of the Mission and of its methods of working, and finally relates the great success that has attended its spiritual ministrations, and its efforts to improve the hygienic condition and the bodily comfort of those employed in deep sea fishing. In a chapter on the "Smacksman's enemy" a description is given of the evils formerly caused by intemperance amongst fishermen, and of the successful contest of the Mission fleet against the foreign coper, the detestable trade of which has quite recently been suppressed by law. The readers of this journal will naturally take special interest in the arrangements that have been made by the Mission for the treatment at sea of fishermen suffering from disease or the results of injury. A vivid account of the hospital ships and of life on board the largest of these is given by Mr. Treves, of the London Hospital, to whom as Chairman of the Hospital Committee of the Mission much credit is due for having organised a very efficient scheme of medical and surgical relief. With this healing work has been associated other means of doing good, such, for instance, as supplying the toilers of our fishing grounds with warm and comfortable clothing, and with books, and inculcating thrift, cleanliness, and temperance. "The Mission," Mr. Gordon points out, "has two sides; it cares for the bodies as well as the souls of the hardy smacksmen," and it is to this combination of practical benevolence with earnest religious ministration that the great success of this movement is mainly due.

* "Home Medicine and Surgery." By Dr. W. J. Mackenzie. (London: L. Upcott Gill. Price 2s. 6d.)

† "What Cheer, O? or, the Story of the Mission to Deep Sea Fishermen." By Alexander Gordon. (London: James Nisbet and Co.)

